# Differential Expression and Function of PDE8 and PDE4 in Effector T cells: Implications for PDE8 as a Drug Target in Inflammation

**DOI:** 10.3389/fphar.2016.00259

**Published:** 2016-08-23

**Authors:** Amanda G. Vang, Chaitali Basole, Hongli Dong, Rebecca K. Nguyen, William Housley, Linda Guernsey, Alexander J. Adami, Roger S. Thrall, Robert B. Clark, Paul M. Epstein, Stefan Brocke

**Affiliations:** ^1^Department of Immunology, University of Connecticut Health CenterFarmington, CT, USA; ^2^Department of Diagnostic Medicine, National Hospital of the Faroe IslandsTórshavn, Faroe Islands; ^3^Department of Cell Biology, University of Connecticut Health CenterFarmington, CT, USA

**Keywords:** cyclic nucleotide phosphodiesterases (PDEs), PDE8A, PDE8 inhibitor, cAMP, T cell, adhesion, allergic airway disease

## Abstract

Abolishing the inhibitory signal of intracellular cAMP is a prerequisite for effector T (Teff) cell function. The regulation of cAMP within leukocytes critically depends on its degradation by cyclic nucleotide phosphodiesterases (PDEs). We have previously shown that PDE8A, a PDE isoform with 40–100-fold greater affinity for cAMP than PDE4, is selectively expressed in Teff vs. regulatory T (Treg) cells and controls CD4^+^ Teff cell adhesion and chemotaxis. Here, we determined PDE8A expression and function in CD4^+^ Teff cell populations *in vivo*. Using magnetic bead separation to purify leukocyte populations from the lung draining hilar lymph node (HLN) in a mouse model of ovalbumin-induced allergic airway disease (AAD), we found by Western immunoblot and quantitative (q)RT-PCR that PDE8A protein and gene expression are enhanced in the CD4^+^ T cell fraction over the course of the acute inflammatory disease and recede at the late tolerant non-inflammatory stage. To evaluate PDE8A as a potential drug target, we compared the selective and combined effects of the recently characterized highly potent PDE8-selective inhibitor PF-04957325 with the PDE4-selective inhibitor piclamilast (PICL). As previously shown, PF-04957325 suppresses T cell adhesion to endothelial cells. In contrast, we found that PICL alone increased firm T cell adhesion to endothelial cells by ~20% and significantly abrogated the inhibitory effect of PF-04957325 on T cell adhesion by over 50% when cells were co-exposed to PICL and PF-04957325. Despite its robust effect on T cell adhesion, PF-04957325 was over two orders of magnitude less efficient than PICL in suppressing polyclonal Teff cell proliferation, and showed no effect on cytokine gene expression in these cells. More importantly, PDE8 inhibition did not suppress proliferation and cytokine production of myelin-antigen reactive proinflammatory Teff cells *in vivo* and *in vitro*. Thus, targeting PDE8 through PF-04957325 selectively regulates Teff cell interactions with endothelial cells without marked immunosuppression of proliferation, while PDE4 inhibition has partially opposing effects. Collectively, our data identify PF-04957325 as a novel function-specific tool for the suppression of Teff cell adhesion and indicate that PDE4 and PDE8 play unique and non-redundant roles in the control of Teff cell functions.

## Introduction

The second messenger cyclic adenosine monophosphate (cAMP) regulates a broad range of biological functions, including the maintenance of immune tolerance (Bourne et al., 1974). cAMP controls the immune response mainly through activation of cAMP-dependent protein kinase A (PKA) which suppresses activation and function of effector T (Teff) cells (Bourne et al., 1974; Baillie et al., 2005; Sitkovsky and Ohta, 2005; Bender and Beavo, 2006; Peter et al., 2007). Recently, we and others have also determined a role for exchange protein activated by cAMP (Epac) in this process (Vang et al., 2013; Almahariq et al., 2015). Formation of site- and function-specific cAMP gradients and spatially distinct signals within cells critically depend on degradation by phosphodiesterases (PDEs), a family of enzymes that hydrolyze cAMP. As a consequence of selective expression and signaling complex formation of PDEs, cAMP signaling is compartmentalized in cells (Baillie, 2009; Houslay, 2010; Conti et al., 2014; Lomas and Zaccolo, 2014). This allows specific PDE isoforms to control distinct cellular functions. Altered expression and positioning of particular PDE isoforms may affect cell and tissue function and lead to pathology. While PDE enzymes are encoded by 21 different genes, 11 gene families (PDEs 1–11) are currently noted based on sequence similarities and biochemical properties and functions (Lerner and Epstein, 2006; Francis et al., 2011; Azevedo et al., 2014; Maurice et al., 2014; Ahmad et al., 2015). Several transcription initiation sites and alternative splicing contribute to the formation of over 100 different forms of PDEs (Lerner and Epstein, 2006; Francis et al., 2011; Azevedo et al., 2014; Maurice et al., 2014; Ahmad et al., 2015).

Based on the unique roles of individual PDEs, selective PDE inhibition by drugs is considered an attractive approach to modulate cell and tissue function. Due to their importance in governing subcellular temporal distribution of cyclic nucleotides, and their accessibility to potent small molecule inhibitors, PDEs make excellent drug targets, including in diseases associated with chronic inflammation (Souness et al., 2000; Burnouf and Pruniaux, 2002; Castro et al., 2005; Martinez and Gil, 2014).

PDE4, PDE7, and PDE8 enzymes are cAMP-specific PDEs expressed in T cells (Lerner and Epstein, 2006). After many years of preclinical development, two novel PDE4 inhibitors have recently been approved for clinical use in chronic obstructive pulmonary disease (COPD) and psoriatic arthritis (Giembycz, 2008; Spina, 2008; Tenor et al., 2011; Poole and Ballantyne, 2014; Genovese et al., 2015). These successes prompted the preclinical development of numerous novel PDE4 inhibitors being tested as potential therapies in a wide range of inflammatory disorders. Since PDEs have different expression and functional profiles in different cell and tissues, a major goal is to selectively inhibit additional PDE families that are expressed in T cells in the hope that distinct and targeted therapeutic activity can be achieved without the side effects associated with PDE4 inhibitors. Previous studies indicated that the high affinity isoforms PDE7A and PDE8A are required for full T cell activation (Li et al., 1999; Glavas et al., 2001). The more recently discovered PDE8 family, encoded by the PDE8A and PDE8B genes, is characterized by high affinity and specificity for cAMP. As we and others have shown, PDE8A is important in immune processes such as T cell activation, effector T cell adhesion, and chemotaxis (Dong et al., 2006; Vang et al., 2010, 2013) as well as breast cancer cell motility (Dong et al., 2015). Until recently, pharmacological approaches to studying PDE8 function have been hampered by the lack of suitable inhibitors. Selective inhibitors of PDE8 enzymes were not available and PDE8 is insensitive to the broad methylxanthine based PDE inhibitors such as 3-isobutyl-1-methylxanthine (IBMX). The broad PDE inhibitor dipyridamole (DP) was the only compound known to inhibit PDE8 enzymes, and its inhibition of these enzymes was somewhat weak (IC_50_ = 4–40 μM; Lerner and Epstein, 2006). In 2010, we were the first to report a potent and selective PDE8 inhibitor developed by Pfizer Inc., PF-04957325, that is now widely used to study PDE8 function *in vitro* and *in vivo* (Vang et al., 2010; Tsai et al., 2011; Tsai and Beavo, 2012; Brown et al., 2013; Demirbas et al., 2013). Our work showed that inhibition of PDE8 with PF-04957325 suppresses two major T cell integrins and firm attachment of effector CD4^+^ T (Teff) cells to endothelial cells (Vang et al., 2010). Further, treatment of mice with PF-04957325 *in vivo* ameliorates the signs of experimental encephalomyelitis without the side effects associated with PDE4 inhibitor treatment (Basole and Brocke, unpublished results).

To further delineate the specific functions of PDE8 selective inhibition in T cells and to explore the therapeutic potential of targeting PDE8, we probed its function by direct comparison of PDE8 inhibition to a PDE4 selective inhibitor with comparable potency, and to analyze PDE8 expression in immune responses *in vivo* utilizing a bi-phasic murine model of ovalbumin (OVA)-induced allergic airways disease (AAD).

## Methods

### Animals

Six to Twelve-week-old female C57BL/6 mice were obtained from Jackson Laboratories (Bar Harbor). Female mice are widely used in experimental allergy and autoimmunity models, and we used them to keep consistency with previous studies (Reinhold et al., 2006; Singh et al., 2008). Experiments were performed according to approved protocols at UConn Health (IACUC Protocol number 100794).

### Bi-phasic model of OVA-induced AAD

For the induction of OVA-induced AAD mice were: (1) sensitized to 25 μg OVA in the adjuvant alum with 3 intraperitoneal injections, 1 week apart; (2) 1 week after the last immunization, mice in each group were exposed to 1% aerosolized OVA in physiological saline (1 h/day, 5 days a week until sacrifice) with an estimated inhaled daily dose of 30–40 μg/mouse as described previously (Yiamouyiannis et al., 1999; Schramm et al., 2004; Singh et al., 2008). Groups of mice (5/group) were sacrificed at 3, 7, and 42 days post start of daily aerosolization. Mice sacrificed at 3 and 7 days represent AAD (peak inflammation) and those at 42 days represent resolution of AAD and the development of tolerance. At sacrifice, the lung draining hilar (mediastinal) lymph node (HLN) and peripheral inguinal lymph nodes (ILN) were dissected and further processed as described below. This bi-phasic model enables us to study the expression of PDE8A during and after acute inflammation.

### Myelin oligodendrocyte glycoprotein (MOG) peptide MOG_35−55_

MOG_35−55_peptide, corresponding to mouse sequence (MEVGWYRSPFSRVVHLYRNGK) was synthesized and purified by the Yale University Synthesis Facility.

### Immunization of mice with MOG_35−55_peptide

Six to Twelve-week-old mice were immunized with MOG_35−55_ in Complete Freund's Adjuvant (CFA; Sigma-Aldrich), a procedure to induce experimental autoimmune encephalomyelitis (EAE) in C57BL/6 mice, an animal model of multiple sclerosis (MS; Preller et al., 2007). A total of 200 μg of MOG_35−55_ peptide and 400 μg of killed *Mycobacterium tuberculosis* (Difco Laboratories) was emulsified in CFA and injected s.c. into the footpads of mice.

### Cell isolation and activation

In the AAD model, lymph node cells (LNC) from HLN and ILN were processed using CD4^+^ T cell isolation kits (Miltenyi Biotec) to separate CD4^+^ from CD4^−^ cell populations. LNC were also dissected from draining popliteal lymph nodes after s.c. immunization with MOG_35−−55_peptide, an autoantigen recognized by T cells in EAE and MS (Preller et al., 2007). Concanavalin A (Con A) activated mouse splenocytes as a source of T cell blasts were prepared and cultured as described (Dong et al., 2006; Vang et al., 2010). Cells were either immediately frozen in appropriate reagents for subsequent qRT-PCR or Western immunoblot analyses or used in proliferation assays as described (Vang et al., 2013).

### RNA isolation and cDNA synthesis

RNA from cells was isolated using the RNeasy mini kit and treated with Turbo DNA-free Dnase (Ambion). cDNA was synthesized using Superscript III reverse transcriptase (Invitrogen; Vang et al., 2010, 2013).

### Quantitative real-time RT-PCR analysis

Quantitative real-time RT-PCR (qRT-PCR) was performed as described previously (Vang et al., 2010, 2013). Ten nanograms of cDNA was amplified by qRT-PCR in a 25 μl reaction using SYBR Green PCR Master Mix (Applied Biosystems). Primers were designed using Primer Express software v3.0. Primers were chosen from gene regions common to all known splice variants of a specific gene product. Primer efficiency was verified by slope analysis to be 100 ± 2.5%. qRT-PCR was performed using an ABI 7500 fast system and data analyzed using the Δ^ct^ method (SDS software v3.0). Primer sequences and amplicon sizes were published previously (Vang et al., 2010, 2013). Expression data were normalized by calculating the ratio of target gene expression/housekeeping gene *rpl19* expression.

### Western immunoblot analysis

Western immunoblot analysis was performed as described previously (Dong et al., 2010; Vang et al., 2013; Almahariq et al., 2015). Mouse T cells were centrifuged at 300 × g for 5 min, washed twice with ice-cold PBS, and lysed in RIPA buffer with 1:100 protease inhibitor cocktail (Sigma). Protein concentration was determined using a BCA Protein Assay Kit (Pierce). Equal amounts of protein were loaded and run on 10% SDS-PAGE gels. Proteins were then transferred onto Immobilon-P transfer membrane (Millipore). Membranes were blocked with 5% BSA in Tris-buffered saline for 1 h at room temperature and probed with primary antibodies overnight at 4°C. Specificity and source of antibodies directed against PDE gene families and isoforms were published previously (Vang et al., 2013). Additionally, a PDE8A specific ab was obtained from Scottish Biomedical and used at a 1:1000–1:2000 dilution on nitrocellulose membrane (Bio-Rad) blots. After probing, membranes were washed three times with TBS-T buffer, and incubated with horseradish peroxidase-conjugated secondary antibody (Anti-Rabbit IgG-horseradish peroxidase was obtained from GE Healthcare or Santa Cruz) at a final dilution of 1:5000 and then washed three more times. Proteins were visualized and quantitated with SuperSignal West Femto Maximum Sensitivity Substrate (Pierce) using Syngene G:Box with GeneSnap BioImaging software. Staining with anti-GAPDH antibody (Abcam) was used for loading control and the signal was used for normalization in quantitation by determining the ratio of the target protein band density/GAPDH band density for CD4^+^ cells divided by the target protein band density/GAPDH band density for the CD4^−^ cell population.

### Adhesion assays

Adhesion assays were performed in 24-well plates with a confluent layer of activated cells of the murine brain endothelium-derived cell line bEnd.3 (ATCC). 100 μM DP, 300 μM IBMX, 1 and 0.1 μM PICL, or PF-04957325 were added to bEnd.3 cells for the last 45 min of TNF-α incubation. T cell blasts or Teff cells were labeled with 5 μM Calcein AM (Molecular Probes) and treated as described above. 7 × 10^5^ pretreated T cell blasts or Teff cells per well were incubated on bEnd.3 cells in RPMI media. After 30 min at 37°C, non-adherent cells were removed by washing with D-PBS. For analysis, 7 × 10^5^ Calcein AM labeled T cell blasts or Teff cells were used as positive controls. Fluorescence was read in a Victor 3v microplate reader (Perkin Elmer) with a fluorescein filter set. The percentage of labeled cells resistant to detachment was calculated as total fluorescence of well divided by fluorescence of 7 × 10^5^ Calcein AM labeled cells.

### Proliferation assays

Isolated Teff cells (5 × 10^4^/well) were cultured in round bottom 96-well plates (Costar) in the presence or absence of soluble anti-CD3 mAb (0.7 μg/ml; R&D). PICL (1, 0.1, 0.01 μM), PF-04957325 (1, 0.1, 0.01 μM), alone or in combination, or vehicle control (0.1% DMSO in media) were added at 0 h (Vang et al., 2013). Proliferation of popliteal LNC in response to MOG_35−55_peptide with inhibitors or vehicle control was performed in round bottom 96-well plates (Costar) at a concentration of 2 × 10^5^ cells/well. After 48 h, 2 μCi per well of [^3^H]thymidine (NEN) was added and cells were harvested 16 h later using a semiautomated cell harvester. [^3^H]thymidine incorporation was determined by scintillation counting.

### Statistics

Experimental groups were compared by analyzing data with the Student's unpaired *t*-test or one-way ANOVA followed by Bonferroni *t*-test using SigmaStat and GraphPad software. Probability levels for statistically significant differences are indicated by the *p*-value in the figure legend and by corresponding asterisks in the figures (^*^p < 0.05, ^**^p < 0.001).

## Results

### Selective expression of PDE8A in CD4^+^ vs. CD4^−^ T cells in inflammation *in vivo*

**Figure 1:**
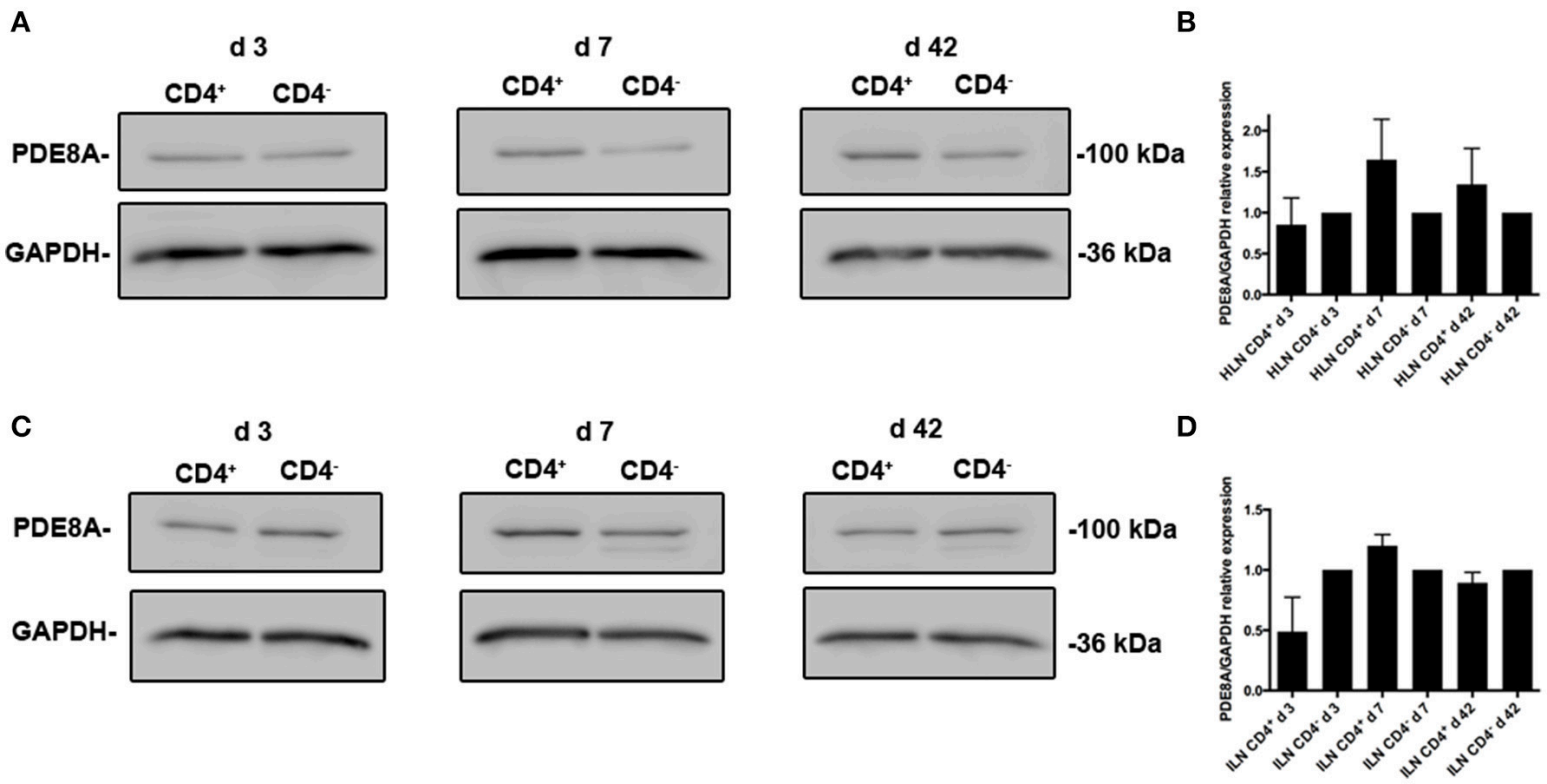
**Differential expression of PDE8A isoforms in CD4^**+**^ and CD4^**−**^ leukocyte populations localized in the HLN of mice with OVA-AAD ***in vivo*****. PDE expression was analyzed by Western immunoblot in *ex vivo* isolated HLN **(A,B)** and ILN **(C,D)** cells from mice with AAD. LNCs were separated into CD4^+^ and CD4^−^ populations by magnetic bead isolation. **A** (HLN) and **C** (ILN) show a comparison of PDE8A protein expression at day 3, 7, and 42 AAD between the CD4^+^ T cell and CD4^−^ leukocyte subpopulations and GAPDH as a loading control for each immunoblot. The data shown are immunoblot analyses from pooled LNCs from 5 HLN that were separated into CD4^+^ and CD4^−^ populations for each day of the experiments. **B** (HLN) and **D** (ILN) show abundance of PDE8A protein determined by immunoblot densitometry and normalized to GAPDH expression. The figure shows the mean + SEM of the quantification of results from HLN samples from days 3, 7, and 42 AAD performed in 2 independent experiments (*n* = 5 mice per group, 2 groups per day, total *n* = 30 mice) as the ratio of the target protein band density/GAPDH band density for CD4^+^ cells divided by the target protein band density/GAPDH band density for the CD4^−^ cell population with the CD4^−^ ratio set at 1.

**Figure 2:**
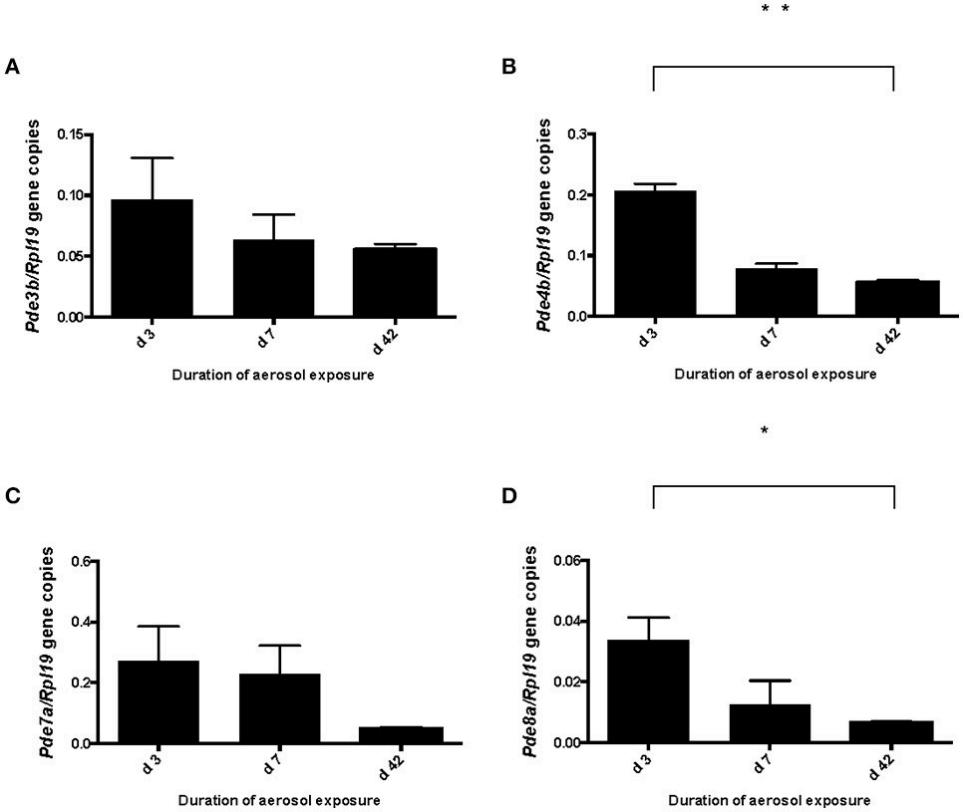
*****Pde3b***, ***pde4b***, ***pde7a***, and ***pde8a*** gene expression in CD4^**+**^ T cells localized in the draining HLN of mice at various days of OVA exposure ***in vivo*****. PDEs were analyzed by qRT-PCR in *ex vivo* isolated HLN cells from mice with AAD separated into CD4^+^ and CD4^−^ cell populations. Data are normalized and expressed as the ratio mean + SEM of target gene expression/housekeeping gene *rpl19* expression. Data in **(A)** show a comparison of *pde3b* gene expression in CD4^+^ cells of HLN samples from days 3, 7, and 42 AAD (*n* = 3). Data in **(B)** show *pde4b* gene expression, in **(C)** *pde7a* gene expression and in **(D)** expression of *pde8a* in CD4^+^ cells of HLN in AAD. (*n* = 5 mice per group, total *n* = 15 mice; ^*^p < 0.05, ^**^p < 0.001, unpaired *t*-test).

We previously determined the expression of PDE8A in Teff and Treg cells *in vitro* and *in vivo* after challenge with antigen (Vang et al., 2010, 2013). Of note, PDE8B expression has not been detected in T cell populations (Hayashi et al., 1998; Dong et al., 2006, 2010). To address the question of whether PDE8 is a potential target for the therapeutic use of selective inhibitors in a T cell mediated inflammatory disease, we analyzed PDE8 expression in lymph nodes of mice challenged with OVA-AAD (Carson et al., 2008). Research over the last three decades has provided evidence that T helper 2 (Th2) CD4^+^ T cells are a major contributor to the development of AAD in animals and asthma in humans. Using a biphasic ovalbumin (OVA)-induced murine model of AAD (Carson et al., 2008), in which resolution occurs with long-term continuous antigen challenge, we separated HLN cells draining the lung tissue at different time points after AAD induction by OVA aerosol exposure (day 3, 7, and 42) into CD4^+^ from CD4^−^ fractions by magnetic bead technique and determined the expression of PDE8A in these cell populations by Western immunoblot. We found that expression of PDE8A protein was higher in CD4^+^ T cells as compared to the CD4^−^ LNC population at day 7 and 42 after AAD induction in HLN (Figures 1A,B). This was not seen in ILN cell populations (Figures 1C,D). Collectively, these data suggest that PDE8A protein abundance is higher in the HLN CD4^+^ T cell population than in the HLN CD4^−^ cell population at the acute intermediate and later stage of AAD. In contrast, in both HLN and ILN, PDE8A protein expression was lower in CD4^+^ T cells as compared to the CD4^−^ LNC population at the early acute stage of AAD on day 3 (Figure 1). Of note, this selective expression pattern was not seen with PDE4B isoforms (data not shown). In contrast to protein expression, the highest of *pde3b, pde4b, pde7a*, and *pde8a* genes in the CD4^+^ T cell fractions from HLN were at day 3 of AAD induction (Figure 2). Taken together, overall expression levels of *pde3b, pde4b, pde7a*, and *pde8a* genes were higher during the acute AAD phase (day 3 or day 7 of the OVA challenge) than at the tolerance (day 42 of OVA challenge) stage of the disease model.

### Opposing effects of PDE8 and PDE4 inhibition on T cell adherence to endothelial cells *in vitro*

**Figure 3:**
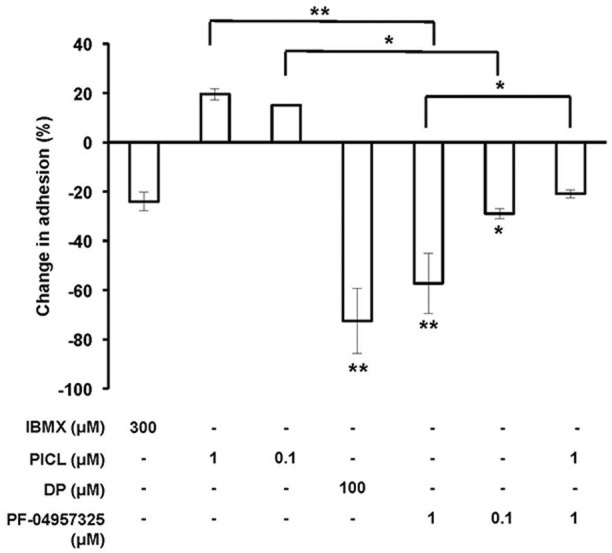
**Inhibiting PDE8 suppresses Teff cell adhesion to endothelial cells and is reversed by PDE4 inhibition**. T cell blasts from C57BL/6 mice and bEnd.3 endothelial cells were incubated alone or in combination with IBMX (300 μM), PICL (1 or 0.1 μM), DP (100 μM) or PF-04957325 (1 or 0.1 μM). Values are normalized to the vehicle condition (0.1% DMSO) and presented as the mean ± SEM percentage of T cell blasts resistant to detachment. Data are averages from three to four independent experiments performed in triplicate (^*^p < 0.05, ^**^p < 0.001, one-way ANOVA and Bonferroni *t*-test).

Functionally, by using the inhibitor DP that inhibits a broad range of PDEs including PDE8 and the recently developed potent and highly PDE8-selective inhibitor PF-04957325 (IC_50_ = 0.0007 μM for PDE8A and < 0.0003 μM for PDE8B), we demonstrated unique effects of PDE8 inhibition on adhesion and chemotaxis of activated T cells (Vang et al., 2010). PDE4 inhibition alone was ineffective in both assay systems. In previous experiments, we repeatedly detected a trend of increase of T cell blast adhesion to endothelial cells and chemotaxis when cells were treated with the highly selective and potent PDE4-selective inhibitor PICL. Therefore, we examined here the effect of combined inhibition of both the PDE4 and PDE8 families which has never been tested. As seen before, DP and PF-04957325 significantly inhibit T cell adhesion in these assays. PF-04957325 had an inhibitory effect on T cell blast adhesion to the endothelial cell line b.End3 by 57% and 29% at 1 μM and 0.1 μM, respectively (Figure 3) (^*^p < 0.05, ^**^p < 0.001; one-way ANOVA and Bonferroni *t*-test). Of note, DP and PF-04957325 were the only compounds that significantly suppressed T cell adhesion. In contrast, the broad PDE inhibitor IBMX—which does not inhibit PDE8—only marginally suppressed adhesion of activated T cells to b.End3 cell. Importantly, PICL, a highly potent PDE4 selective inhibitor, reversed the inhibitory effect of PF-04957325 at 1 μM from 57 to 21% when used in combination (Figure 3; ^*^p < 0.05; one-way ANOVA and Bonferroni *t*-test). These results clearly establish opposing effects, including partial reversal, of PDE8 vs. PDE4 inhibition on rapid T cell adhesion *in vitro*, a conclusion which is additionally supported by PICL enhancing adhesion to 21% above the DMSO control when acting alone (Figure 3; ^*^p < 0.05; one-way ANOVA and Bonferroni *t*-test).

### Differential potency of PDE8 and PDE4 inhibition on T cell proliferation *in vitro* and *ex vivo*

**Figure 4:**
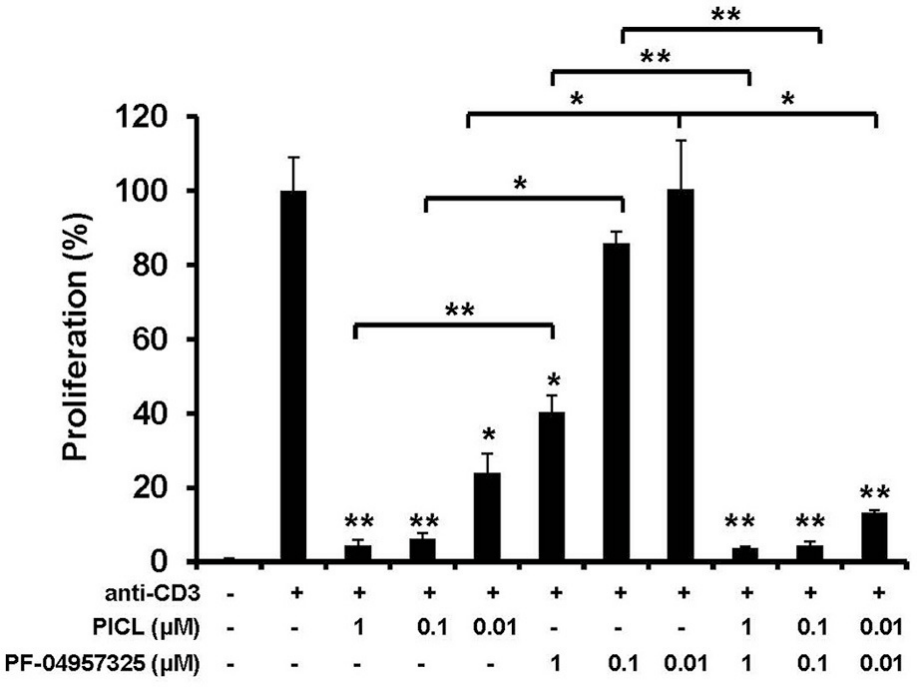
**Selective inhibition of Teff cell proliferation by PDE4 inhibition ***in vitro*****. Proliferation of purified CD4^+^CD25^−^ Teff cells exposed to PDE inhibitors. Teff cells (5 × 104/well) were cultured with immobilized anti-CD3 mAb or control in the presence of IBMX (300 μM), DP (100 μM), PICL, PF-04957325 alone or in combination, or vehicle control (0.1% DMSO). The extent of proliferation was determined by [^3^H]thymidine incorporation at 64 h and results are presented as mean + SEM counts per min (cpm). Data are representative of three to five independent experiments performed in triplicate (^*^p < 0.05, ^**^p < 0.001, comparisons to vehicle were analyzed using a one-way ANOVA and Bonferroni *t*-test).

Our results on adhesion are notable since in proliferation studies, PICL was significantly more efficient at suppressing Teff cell proliferation compared to PF-04957325 indicating a selective effect of PDE8 inhibition on rapid T cell adhesion to endothelial cells. To further probe the selectivity of PDE8 action in the control of T cell function, we examined the single and combined effect of broad and selective inhibitors on purified Teff cell proliferation in response to polyclonal or antigen-specific stimulation through the T cell receptor (TCR) (Figure 4). Isolated Teff cells were stimulated with immobilized anti-CD3 mAbs in the presence of broad and selective PDE inhibitors over a range of concentrations alone and in combination in order to establish a dose-response. The PDE4-selective inhibitor PICL was over 100-times more effective in suppressing Teff cell proliferation than PF-04957325 (compare 1 μM PF-04957325 vs. 0.01 μM PICL, Figure 4). There was a slight additional effect when both inhibitors were combined, whereas the opposing effects seen in the adhesion assays (Figure 3) were not observed in any of the proliferation experiments (Figure 4).

**Figure 5:**
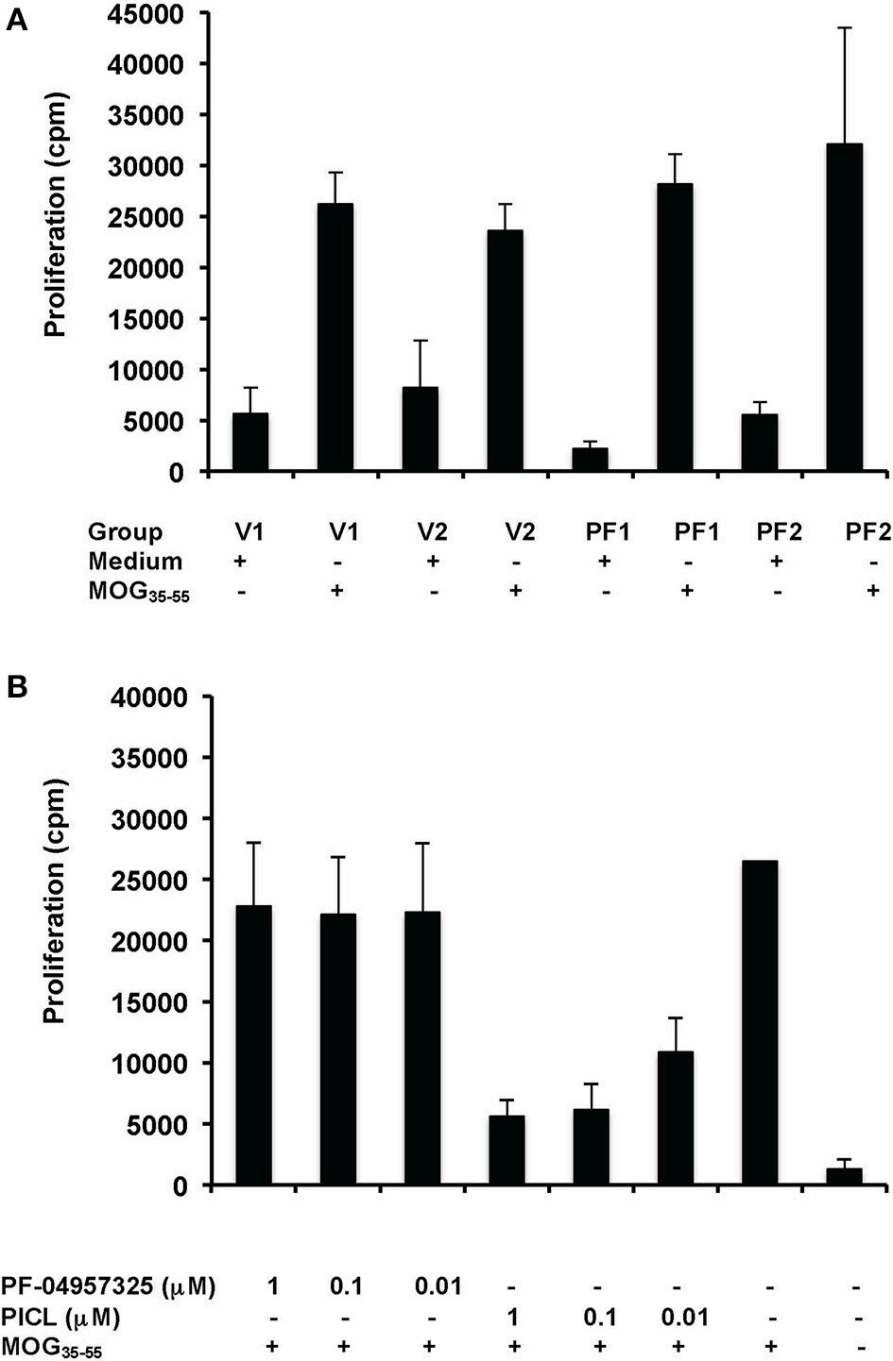
**PF-04957325 does not suppress T cell proliferation in response to MOG_**35−55**_***ex vivo*** and ***in vitro***. (A)** C57BL/6 mice were immunized with MOG_35−55_ and CFA and treated twice daily from days 8 to 10 by subcutaneous administration of PF-04957325 (PF1, PF2) or vehicle control (V1, V2) (*n* = 2 mice per group, total *n* = 4). Each injection contained a dose of 2.5 mg/kg PF-04957325 dissolved in 100 μl vehicle (PF-04957325) or 100 μl vehicle alone (vehicle control). **(B)**. C57BL/6 mice were immunized with MOG_35−55_ and CFA (*n* = 2). **(A,B)** Draining popliteal lymph nodes were dissected 10 day after immunization and an *in vitro* proliferation assay was performed under conditions as indicated. MOG_35−55_ (50 μg/ml) was present where indicated **(A,B)**, together with PDE inhibitors *in vitro* as shown **(B)**.

Additionally, we tested *in vitro* recall stimulation of T cells from lymph nodes of mice immunized with an encephalitogenic peptide, MOG_35−55_, of the myelin antigen MOG which is an autoantigen in EAE and MS (Brocke et al., 1996; Preller et al., 2007). In these assays, in contrast to experiments with anti-CD3 stimulation, antigen presenting cells are present during the entire experiment. As shown in Figure 5, proliferation was not inhibited by PF-04957325 application *in vivo* (Figure 5A) or *in vitro* (Figure 5B). In contrast, PICL profoundly inhibited the proliferation in response to MOG_35−55_ *in vitro* (Figure 5B), similar to the effect seen in anti-CD3 responses (Figure 4).

## Discussion

PDE enzymes are highly successful drug targets for treating vascular and inflammatory diseases (Martinez and Gil, 2014; Maurice et al., 2014). The ability to form site- and function-specific cAMP gradients within the cell critically depends on its degradation by PDEs which are pivotal regulators of intracellular cAMP activity (Baillie, 2009; Houslay, 2010; Conti et al., 2014; Lomas and Zaccolo, 2014). Observations that inhibition of PDE4, an abundantly expressed PDE in T cells, blocks T cell activation and function through elevating cAMP, prompted the development of PDE4 inhibitors as potential immunosuppressive therapies (Ekholm et al., 1997; Lerner and Epstein, 2006; Lugnier, 2006; Giembycz, 2008; Spina, 2008). After years of research and development of numerous candidate compounds, the FDA approval of the PDE4 inhibitors roflumilast and apremilast in 2011 and 2014 for the treatment of COPD and psoriatic arthritis represent important breakthroughs for the use of PDE inhibitors in the therapy of human inflammatory disorders. Due to the limitations of PDE4 inhibitors set by their narrow therapeutic window, several alternative strategies are pursued to target PDEs in immune diseases. These include the inhibition of different cAMP-specific PDEs, such as PDE7 and PDE8. The recent development of the new PDE8 inhibitor PF-04957325 has helped to identify PDE8 as a novel target for suppression of effector T cell functions due to the important role of the PDE8 family in regulating cAMP signaling in these cells (Martinez and Gil, 2014). After the initial observation that PDE8A is expressed in T cells, several reports documented the role of PDE8 in controlling T cell and cancer cell motility (Glavas et al., 2001; Dong et al., 2006, 2015; Vang et al., 2010, 2013). Together, PDE7 and PDE8 are now seen as new emerging targets to treat inflammation (Martinez and Gil, 2014). Our data demonstrate for the first time robust PDE8A expression in leukocytes associated with an inflammatory disease *in vivo*, a mouse model of AAD. The preferential expression of PDE8A protein in the CD4^+^ T cell subset during the acute AAD stage and its subsequent recession in the non-inflammatory tolerant stage, together with the common assumption that CD4^+^ Teff cells are a major contributor to the development of AAD in animals and asthma in humans, strengthen the case to further examine PDE8A inhibition in preclinical and clinical studies of inflammatory disorders, including human respiratory airway diseases.

Previously, we failed to detect any suppressive effect of the highly potent PDE4-selective inhibitor PICL on T cell adhesion to activated endothelial cells. In contrast, DP reduced adhesion of T cell blasts by 73% while PF-04957325 reduced adhesion by a maximum of 53%. However, PICL was also very efficient at suppressing proliferation. Thus, our data suggest that a rapid effect on T cell adhesion critically depends on a PDE inhibitor that blocks PDE8 enzymatic activity, while inhibition of Teff cell proliferation is less dependent on blocking the PDE8 isoform. In this present study, we explored the precise action of PDE8 and PDE4 selective inhibition of T cell adhesion by testing inhibitors over a range of concentrations and in combination. In doing so, we found an entirely novel effect of PDE4 inhibition enhancing adhesion of T cells to endothelial cells and opposing the inhibitory effect of PDE8 inhibition. These data suggest distinct signaling pathways utilized by PDE8 and PDE4 in T cells, a hypothesis further supported by the differential action of selective inhibitors of these enzymes in proliferation assays.

At present, it is unknown what accounts for the different effects of selected PDE isoform inhibition during adhesion and proliferation. Regulation of adhesion of leukocytes to vascular endothelial ligands is a very fast process measured in microseconds (Grabovsky et al., 2000). A possible mechanism may be that DP and PF-04957325 upregulate intracellular cAMP levels more rapidly and efficiently than PDE inhibitors that do not block PDE8, requiring a longer time of action for less efficient PDE inhibitors during Teff cell adhesion (Zhuplatov et al., 2006). Since PDE8A is a very high affinity cAMP-specific PDE with a *K*m value ranging from 0.04 to 0.15 μM, 40–100 times lower than that of PDE4, it is likely to be functioning at lower cAMP concentrations than PDE4 and may thus be involved in the control of intracellular cAMP concentrations at basal levels and in the immediate response to acute increases of cAMP in specific cell regions (Fisher et al., 1998; Soderling et al., 1998; Vasta, 2007). This mechanism would be consistent with our data. Major mechanistic insights into PDE8A signaling came from a recent report that PDE8A associates with Raf-1 to protect it from inhibitory phosphorylation by PKA (Brown et al., 2013). Raf kinases have been shown to regulate integrin α4β1-mediated T cell resistance to shear stress which may explain our observations in T cell adhesion assays (Brown et al., 2014).

We also analyzed the effects of PF-04957325 administration on CD4^+^ responses in draining lymph nodes 10 days after MOG_35−55_ and CFA immunization. We found no effect of PF-04957325 administered s.c. on CD4^+^ Teff cell proliferation (Figure 5) or production of IFNγ or IL-17, nor changes in percentage and numbers of CD4^+^, Foxp3^+^ (Treg cells), γδTCR^+^ or Ki-67^+^ (proliferating) T cells in the draining lymph nodes of CFA and MOG_35−55_ immunized mice (data not shown). Additionally, in contrast to the PDE4-selective inhibitor PICL, PF-04957325 did not significantly suppress T cell proliferation *in vitro* in response to MOG_35−55_ and showed over 100-times lower efficacy in suppressing proliferative responses to anti-CD3 stimulation. The different potency of PF-04957325 in assays using whole lymph nodes could indicate a role for costimulation provided by antigen-presenting cells overcoming its moderate anti-proliferative action when isolated Teff cell proliferation were stimulated by anti-CD3 mAb. Overall, our results indicate a non-redundant role for PDE8 in regulating T cell adhesion to vascular endothelium through the cAMP signaling pathway. The data further suggest that PDE8 inhibition, if successful *in vivo* in inflammatory diseases, may selectively target leukocyte motility without exerting global immunosuppressive effects on cytokine production and cell proliferation and thus provide a highly selective therapeutic tool while maintaining the proven characteristics of PDE inhibitors as successful drugs. Taken together, efforts to develop and test selective inhibitors of PDE8 such as PF-04957325 should be undertaken as a means to develop novel therapeutic agents for treatment of inflammatory disorders mediated by activated T cells (Steinman, 1996, 2004; Ford et al., 2003; Ransohoff, 2007; Li and Ransohoff, 2008).

## Author contributions

AV performed experiments, summarized and analyzed data and wrote the manuscript. CB, HD, RN, WH, LG, and AA performed experiments and reviewed and edited the manuscript. RT and RC reviewed and edited the manuscript. SB and PE designed the experiments and reviewed and wrote the manuscript. SB and RC performed some of the experiments.

### Conflict of interest statement

The authors declare that the research was conducted in the absence of any commercial or financial relationships that could be construed as a potential conflict of interest.
